# The Prognostic Value of Mitotic Activity Index (MAI), Phosphohistone H3 (PPH3), Cyclin B1, Cyclin A, and Ki67, Alone and in Combinations, in Node-Negative Premenopausal Breast Cancer

**DOI:** 10.1371/journal.pone.0081902

**Published:** 2013-12-04

**Authors:** Marie Klintman, Carina Strand, Cecilia Ahlin, Sanda Beglerbegovic, Marie-Louise Fjällskog, Dorthe Grabau, Einar Gudlaugsson, Emiel A. M. Janssen, Kristina Lövgren, Ivar Skaland, Pär-Ola Bendahl, Per Malmström, Jan P. A. Baak, Mårten Fernö

**Affiliations:** 1 Department of Clinical Sciences, Division of Oncology, Lund University, Lund, Sweden; 2 Skåne Department of Oncology, Skåne University Hospital, Lund, Sweden; 3 Department of Oncology, Örebro University Hospital, Örebro, Sweden; 4 Division of Pathology, Växjö Central Hospital, Växjö, Sweden; 5 Department of Clinical Sciences, Division of Oncology, Radiology, and Clinical Immunology, Uppsala University, Uppsala, Sweden; 6 Department of Clinical Sciences, Division of Pathology, Lund University and Skåne University Hospital, Lund, Sweden; 7 Department of Pathology, Stavanger University Hospital, Stavanger, Norway; University Medical Centre Utrecht, Netherlands

## Abstract

Proliferation, either as the main common denominator in genetic profiles, or in the form of single factors such as Ki67, is recommended for clinical use especially in estrogen receptor-positive (ER) patients. However, due to high costs of genetic profiles and lack of reproducibility for Ki67, studies on other proliferation factors are warranted. The aim of the present study was to evaluate the prognostic value of the proliferation factors mitotic activity index (MAI), phosphohistone H3 (PPH3), cyclin B1, cyclin A and Ki67, alone and in combinations. In 222 consecutive premenopausal node-negative breast cancer patients (87% without adjuvant medical treatment), MAI was assessed on whole tissue sections (predefined cut-off ≥10 mitoses), and PPH3, cyclin B1, cyclin A, and Ki67 on tissue microarray (predefined cut-offs 7th decile). In univariable analysis (high *versus* low) the strongest prognostic proliferation factor for 10-year distant disease-free survival was MAI (Hazard Ratio (HR)=3.3, 95% Confidence Interval (CI): 1.8-6.1), followed by PPH3, cyclin A, Ki67, and cyclin B1. A combination variable, with patients with MAI and/or cyclin A high defined as high-risk, had even stronger prognostic value (HR=4.2, 95%CI: 2.2-7). When stratifying for ER-status, MAI was a significant prognostic factor in ER-positive patients only (HR=7.0, 95%CI: 3.1-16). Stratified for histological grade, MAI added prognostic value in grade 2 (HR=7.2, 95%CI: 3.1-38) and grade 1 patients. In multivariable analysis including HER2, age, adjuvant medical treatment, ER, and one proliferation factor at a time, only MAI (HR=2.7, 95%CI: 1.1-6.7), and cyclin A (HR=2.7, 95%CI: 1.2-6.0) remained independently prognostic. In conclusion this study confirms the strong prognostic value of all proliferation factors, especially MAI and cyclin A, in all patients, and more specifically in ER-positive patients, and patients with histological grade 2 and 1. Additionally, by combining two proliferation factors, an even stronger prognostic value may be found.

## Introduction

To avoid overtreatment of low-risk early breast cancer patients, and at the same time justify dose-intensive treatments for high-risk patients, better tools are needed when estimating risk and deciding on adjuvant medical treatment. Studies on genetic profiles, where the main common denominator is proliferation genes [1], have identified groups with prognostic differences specifically within estrogen receptor (ER) positive disease [2–5], and patients with histological grade 2 [6,7]. These profiles are to some extent recommended for clinical use [8]. A recent study has also suggested that although all commercially available genetic profiles add prognostic information in lymph-node negative patients, the best prediction of recurrences was found when combining different genetic profiles [9]. There are however yet no published prospective studies to support their use, and the cost of these profiles is still substantial. In the 2011 St Gallen guidelines, the proliferation factor Ki67 is now instead recommended for use for approximation of the biological intrinsic subtypes identified by genetic arrays [10]. More specifically, proliferation is used to distinguish between the “luminal A”- and “luminal B”-like subtypes. There is however still no consensus on how to assess Ki67, or which cut-off to choose, and international multicenter reproducibility studies are lacking, which limits the clinical value of Ki67 [11]. A recent study showed that subjective counts of Ki67 is poorly reproducible even when assessed by experienced pathologists, and inferior to digital image analysis (DIA) [12]. However subjective counts are still most commonly used. The strong prognostic value of the proliferation factor mitotic activity index (MAI) has been shown in a number of publications [13–19], even prospective studies [20,21]. There have been questions as to the reproducibility of MAI [22,23], but, when adhering to the recommended guidelines, MAI is highly reproducible [14,20,24]. Phosphohistone H3 (PPH3) is a protein involved in chromatin condensation and decondensation and is present in the G2 to M transition [25,26]. PPH3 has been shown to have a strong prognostic value in lymph-node negative breast cancer, and the clear and contrast-rich PPH3 staining has an advantage of being easily assessed with high inter-observer reproducibility [27–29]. Cyclin B1 regulates onset of mitosis, and high levels of cyclin B1 in breast cancers has in several studies been shown to be a negative prognostic marker [30–33]. High levels of the S-phase specific cyclin A, is also associated with a worse outcome in breast cancer [34–36]. 

The aim of the present study on premenopausal node-negative breast cancer patients was to investigate the prognostic value of the proliferation factors MAI, PPH3, cyclin B1, cyclin A, and Ki67. Secondly, this study aimed at investigating whether the prognostic value was dependent on ER-status and histological grade, and if the prognostic value of proliferation is strengthened when two proliferation factors are combined.

## Material and Methods

### Ethics Statement

The study was approved by the ethics committee of Lund University Hospital (LU 240-01). The study protocol contained a written patient information sheet which was given to all patients and a written instruction for the doctor on how the information should be given to the patients. This was followed by verbal informed consent which was documented in the patients´ records. Written informed consent was not required by the Ethics Committee of Lund when this study was conducted, and the above mentioned procedure was preferred in national trials and approved by the ethics committee of Lund University Hospital prior to the initiation of the study.

### Patients

The initial patient population consists of 237 node-negative premenopausal patients who from 1991-1995 had been included in a prospective study on the prognostic value of S-phase fraction [37]. In total 222 patients were included in this study. In 14 cases no paraffin blocks were available at the pathology departments, and the remaining loss is specified below separately for each proliferation factor. Detailed information on primary surgery, adjuvant radiotherapy and adjuvant medical treatment have been described earlier [37], and patient and tumour characteristics can be found in Table 1. 

**Table 1:** Characteristics of 222 premenopausal patients with node-negative breast cancer.

**Age, years**	Median	47
	Range	30-57
**Tumour size, mm**	Median	15
	Range	5-45
**No. of lymph nodes removed**	Median	9
	Range	0-42*
**Primary treatment, n**	Breast conserving surgery without radiotherapy	57
	Breast conserving surgery + postoperative radiotherapy	106
	Modified radical mastectomy without radiotherapy	52
	Modified radical mastectomy + postoperative radiotherapy	7
**Adjuvant medical treatment, n**	All	29
	Adjuvant endocrine treatment	8
	Tamoxifen 20 mg daily for 5 years	7
	Oophorectomy	1
	Adjuvant chemotherapy (CMF i.v. nine cycles**)	21
**Local/locoregional recurrence** *** **, No. of patients**	≤ 10 years	32
**Distant metastases , No. of patients**	≤ 10 years	48
**DDFS, % (95% CI)**	10 years	78 (72-83)
**Overall survival, % (95% CI)**	10 years	80 (74-84)

^*^ One patient with axillary exeresis and no identified lymph nodes.

^**^ CMF, cyclophosphamide, methotrexate, and 5-fluoruracil.

^***^ diagnosed as only events, or before distant recurrences

The median follow-up was 10.8 years for the end-point distant disease-free survival (DDFS) for patients alive and free from distant metastases at the last review of the patients´ records. Data from the first 10 years after diagnosis are presented. Whenever applicable, the REMARK recommendations for reporting of tumour marker studies were followed [38]. 

### Histological grading, ER and progesterone receptor (PR) analyses

Tumour grading was performed according to Elston and Ellis and as previously described [37,39]. ER and PR status were analyzed by enzyme immunoassay (EIA) on cytosol samples as previously described [37].

### Human epidermal growth factor receptor 2 (HER2) status

HER2 protein was analyzed as previously described [40]. Amplified tumours and tumours with Herceptest 3+ in which fluorescent *in situ* hybridization analysis was non-evaluable were considered HER2-positive. 

### MAI

MAI was assessed by one experienced pathologist (JB) on lightly stained haematoxylin-eosin whole sections according to the MMMCP 1987 protocol [41]. The mitotic figures were carefully defined to avoid inclusion of apoptotic and necrotic cells. At low magnification, the area with subjectively the highest proliferation in the periphery of the tumour with invasive cancer, no necrosis or extensive inflammation, with an invasive component of at least 3 mm diameter was identified. Starting from this area, structures that were undeniable mitotic figures were identified (if necessary after focusing up and down) and counted at 400x magnification (objective 40x, field diameter 450 µm at specimen level) in 10 consecutive fields of view (FOV). Cases with thick or poorly fixed/stained sections (n=1), with extensive cancer in situ (CIS) or inflammation (n=8), or with an invasive area of <3 mm diameter (n=5) were excluded. In 14 cases, there were no available H&E sections left for evaluation. The MAI is defined as the total number of mitoses in an area in the section of 1.59 mm^2^. The same cut-off as in previous publications was chosen, with ≥10 mitoses defined as high risk [20,24]. In all cases with MAI values between 5 and 15, a second assessment was later performed without knowledge of the results of the first assessment, and the highest value was chosen. In case of discrepancies of >3 mitoses, a third measurement was performed, and the highest value of the two assessments closest to each other was chosen for further analysis. Data on MAI was available for 195 patients. 

### Tumour Tissue Microarray (TMA) for assessment of cyclin B1, Ki67, cyclin A, and PPH3

The TMA was constructed as previously described with two 0.6 mm cores available for stainings for Ki67, cyclin B1, cyclin A, and two 1 mm cores for PPH3 [40]. Cores were taken from representative areas of the tumour, mainly from the periphery, but also from more central parts of the tumour. 

### Cyclin B1, Ki67 and cyclin A

Assessment of cyclin B1, Ki67, and cyclin A were all done in high-power fields (40X obejctive) using a light microscope. Cyclin B1 was assessed as previously described [30]. Antigen retrieval was performed in Tris-EDTA pH 9 buffer in a pressure cooker for 4 minutes at 121°C. Slides were stained with a cyclin B1 antibody diluted 1:200 (rabbit monoclonal cyclin B1 1495-1, Epitomics Inc Burlingame, CA, USA) in an Auotstainer (DakoCytomation) for 30 minutes at room temperature. Diaminobenzidene (DAB) was used as chromogen, and 200 tumour cells were manually counted by two investigators. 5 cases were excluded, as there were fewer than 200 tumour cells in the TMAs. The level of agreement between the two readers was good (correlation coefficient 0.91 between estimated proportions and kappa value 0.77 when applying the cut-off defined below to both series), and results from only one of the readers was chosen for further analysis. Data on cyclin A and Ki67 was already available and assessments and reasons for exclusion have been described previously [34,40]. For cyclin A 200 cells were counted manually by two investigators. The level of agreement between the two readers was good (kappa value 0.71), and the results from the more experienced of the two investigators was chosen for further analyses. Four cases were excluded as there were no or less than 200 cancer cells in the TMA. Ki67 had also been assessed previously by three independent readers. A senior pathologist used a semiquantitative approach, the other two readers manual counting of all tumour cells in a TMA core. The level of agreement between readers was found to be good (kappa values of 0.83-0.88), and the semiquantitative assessments were chosen for further analyses. 23 cases were excluded due to staining difficulties (n=16) or loss of individual tumour sections in the TMA (n=7). The 7^th^ decile was pre-defined as cut-off, as in previous publications, which for cyclin B1 corresponded to >12.5% positively stained cells [30], cyclin A >15% [36], and Ki67 >20% positively stained cells [40]. Data was available for 217, 218, and 199 patients for cyclin B1, cyclin A, and Ki67, respectively. 

### PPH3

Antigen retrieval was performed in Tris-EDTA buffer (pH 9.0) and heated for 3 minutes at 110°C, followed by 10 minutes at 95°C and then cooled to 20°C. Slides were stained in a Dako Autostainer. The rabbit polyclonal anti-phosphohistone H3 (ser 10 Upstate #06-570, Lake Placid, NY, USA) at 1:1500 dilution was used and incubated for 60 minutes at 22°C. DAB was used as chromogen. Assessment of PPH3 was done under a light microscope in high power fields (40X objective). All positively stained nuclei in the invasive tumour in a TMA core were counted, disregarding nuclei with fine granular staining as they are not in the G2 phase. Similar to assessment of MAI, previous assessments of PPH3 have been performed on whole tissue sections, starting at the periphery of the tumour on 10 consecutive FOVs with a total area of 1.59 mm^2^ [28]. Therefore in the present study the number of positively stained nuclei in one TMA core was multiplied with 1.59 and divided by the total area the TMA, 1.13mm^2^. The 7^th^ decile was chosen as pre-defined cut-off, the same distribution as for the other proliferation factors assessed on TMA, which corresponded to ≥7 positive cells. Two cases were excluded, as there were no tumour cells in the TMA. Therefore data on PPH3 was available for 221 patients

### Statistics

The primary end-point was 10-year distant disease-free survival (DDFS). The Kaplan Meier method was used for estimation of DDFS, and the log-rank test for comparing survival in different strata. The Cox proportional hazards model was chosen for estimation of univariable- and multivariable hazard ratios (HR). Proportional hazards assumptions were checked graphically and by Schoenfeld´s test [42]. All factors were used as dichotomous covariates in the statistical analyses, with the exception of grade (three groups) and age, which was also analysed as a continuous variable. The null hypothesis of no prognostic effect by the different proliferation factors in ER-positive and ER-negative patients was evaluated using a Cox model with a term for the interaction between ER-status and the proliferation factor. Cut-off values were chosen before statistical analyses. Pearson's correlation coefficient (*r*), Pearson's χ^2^ test, and for histological grade Pearson's χ^2^ test for trend, were used for analyses of associations between factors. Kappa statistics were used to evaluate the agreement between readers regarding cyclin A, cyclin B1, and Ki67 status.

All *P*-values corresponded to two-sided tests and *p*<0.05 was considered significant. The statistical calculations were performed using Stata version 12.1 (StataCorp 2012, College Station, TX, USA).

## Results

### Patient and tumour characteristics

During the first 10 years after diagnosis 32 patients had locoregional recurrences (as only events or diagnosed before distant metastases), 48 patients had distant recurrences, and 45 deaths were recorded (43 of breast cancer). The 10-year DDFS for all patients was 78% (95% confidence interval (CI): 72-83%), and 10-year overall survival (OS) was 80% (95%CI: 73-84%). Detailed patient characteristics can be found in Table 1. All proliferation factors were strongly correlated (*r*: 0.44-0.74), Table 2. High MAI, PPH3, cyclin B1, Ki67, and cyclin A were significantly associated with younger age, larger tumour size, ER-negativity, HER2-positivity, and high grade (data not shown).

**Table 2:** Correlation coefficients (*r*) between the five different proliferation factors MAI, PPH3, cyclin A, Ki67, and cyclin B1.

		**MAI**	**PPH3**	**cyclin A**	**Ki67**	**cyclin B1**
**MAI**	*r*	1.00				
	No of patients	195				
**PPH3**	*r*	0.59	1.00			
	No of patients	194	221			
**cyklin A**	*r*	0.74	0.53	1.00		
	No of patients	193	217	218		
**Ki67**	*r*		0.45	0.72	1.00	
	No of patients	173	198	197	199	
**cyklin B1**	*r*	0.62	0.44	0.67	0.67	1.00
	No of patients	191	216	213	195	217

### Distant disease-free survival at 10 years

The analyses presented below are based on all the 222 patients in the study or on subsets based on ER-status or histological grade. Similar results, but generally stronger, were found when the 29 patients (13%) who had received any adjuvant medical treatment were excluded (data not shown).

### Univariable analyses

**Figure 1:**
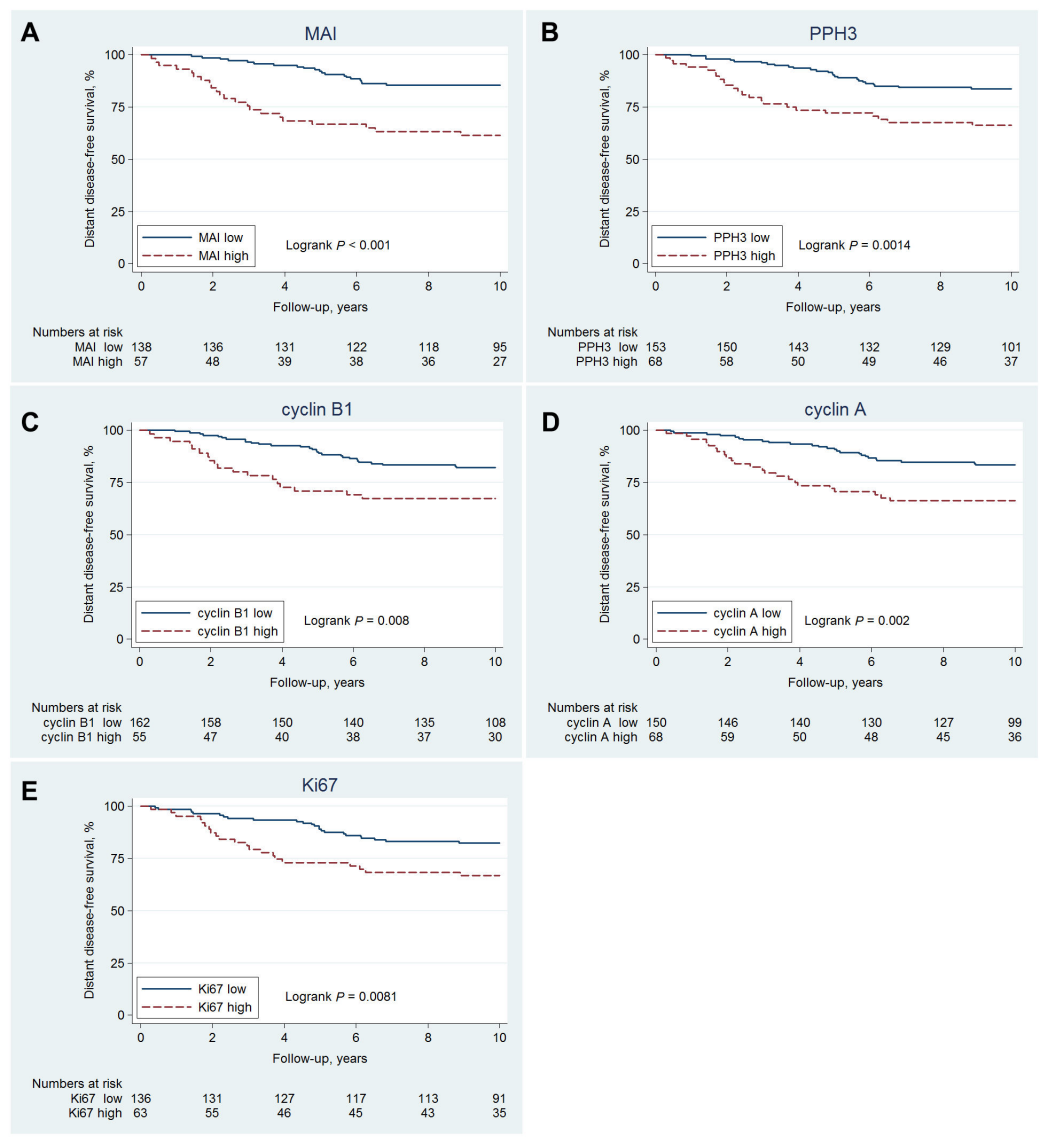
10-year distant disease-free survival of premenopausal women with lymph-node negative breast cancer according to (a) MAI-status (b) PPH3-status, (c) cyclin B1-status, (d) cyclin A-status, and (e) Ki67-status.

In univariable analysis MAI (high: ≥10 *versus* low: <10) was the strongest proliferation factors for DDFS (HR=3.3, 95%CI 1.8-6.1, *p*<0.001), corresponding to a 10-year DDFS of 61% (95%CI: 48-73%) and 86% (95%CI: 78-90%) for high- and low-risk patients, respectively. This was followed by PPH3 (HR=2.4, 95%CI 1.4-4.3, *p*=0.002), cyclin A, Ki67, and cyclin B1, Table 3, Figure 1 a-e. HER2, PR, and age were also significant prognostic factors, but ER and tumour size were not, Table 3 *.*

**Table 3:** Univariable analysis of prognostic factors for 10-year distant disease-free survival for all 222 premenopausal patients with node-negative breast cancer (left), and for the ER-positive patients only (right).

		**All patients (n=222, 48 events)**						**ER-positive patients (n=148, 27 events)**					
**Factor**		**n**	**Distant recurrence**		**Hazard Ratio**	**95% Confidence Interval**	***p*-value**	**n**	**Distant recurrence**		**Hazard Ratio**	**95% Confidence Interval**	***p*-value**
			**No**	**%**					**No**	**%**			
**Age, years**	All	222	48	22	0.91	0.87-0.95	<0.001	148	27	18	0.89	0.84-0.95	<0.001
**Age, years**	All	222											
	>50	55	4	7	1.0			43	1	2	1.0		
	≤50	167	44	26	4.1	1.5-11	0.007	105	26	25	12	1.7-90	0.014
**Tumour size, mm**	All	222											
	≤20	66	33	20	1.0			116	21	18	1.0		
	>20	156	15	27	1.5	0.80-2.7	0.21	32	6	19	1.0	0.42-2.6	0.92
**Histological grade**	All	217											
	1	69	10	14	0.37	0.18-0.78	0.009	57	7	12	0.25	0.09-0.65	0.005
	2	79	15	19	0.50	0.26-0.97	0.039	62	10	16	0.33	0.14-0.79	0.001
	3	69	23	33	1.0			25	10	40	1.0		
**ER**	All	222											
	Pos	148	27	18	1.0								
	Neg	74	21	28	1.7	0.98-3.1	0.059						
**PR**	All	222											
	Pos	160	28	18	1.0			137	25	18	1.0		
	Neg	62	20	32	2.2	1.2-3.9	0.008	11	2	18	1.1	0.26-4.6	0.91
**HER2**	All	209											
	Neg	186	32	17	1.0			131	23	18	1.0		
	Pos	23	11	48	3.9	2.0-7.8	<0.001	11	4	36	2.7	0.92-7.7	0.070
**MAI**	All	195											
	Low	138	20	14	1.0			117	15	13	1.0		
	High	57	22	39	3.3	1.8-6.1	<0.001	18	10	56	7.0	3.1-16	<0.001
**PPH3**	All	221											
	Low	153	25	16	1.0			107	15	14	1.0		
	High	68	23	34	2.4	1.4-4.3	0.002	41	12	29	2.4	1.1-5.2	0.029
**Cyclin B1**	All	217											
	Low	162	29	18	1.0			127	20	16	1.0		
	High	55	18	33	2.2	1.2-3.9	0.010	16	6	38	2.9	1.2-7.2	0.023
**Cyclin A**	All	218											
	Low	150	25	17	1.0			121	18	15	1.0		
	High	68	23	34	2.4	1.3-4.2	0.003	24	9	38	3.1	1.4-7.0	0.001
**Ki67**	All	199											
	Low	136	24	18	1.0			110	16	15	1.0		
	High	63	21	33	2.2	1.2-3.9	0.010	22	10	45	3.9	1.8-8.6	0.001
**MAI and/or PPH3 high**	All	194											
	Low	118	16	14	1.0			101	12	12	1.0		
	High	76	26	34	3.0	1.6-5.7	<0.001	34	13	38	4.2	1.9-9.1	<0.001
**MAI and/or cyclin B1 high**	All	191											
	Low	125	17	14	1.0			106	13	12	1.0		
	High	66	24	36	3.3	1.8-6.1	<0.001	25	11	44	4.8	2.2-11	<0.001
**MAI and/or cyclin A high**	All	193											
	Low	125	15	12	1.0			107	12	11	1.0		
	High	68	27	40	4.2	2.2-7.9	<0.001	26	13	50	6.4	2.9-14	<0.001
**MAI and/or Ki67 high**	All	173											
	Low	108	15	14	1.0			95	12	13	1.0		
	High	65	25	38	3.4	1.8-6.5	<0.001	24	12	50	5.5	2.5-12	<0.001

### The prognostic value of proliferation stratified for ER-status and histological grade

**Figure 2:**
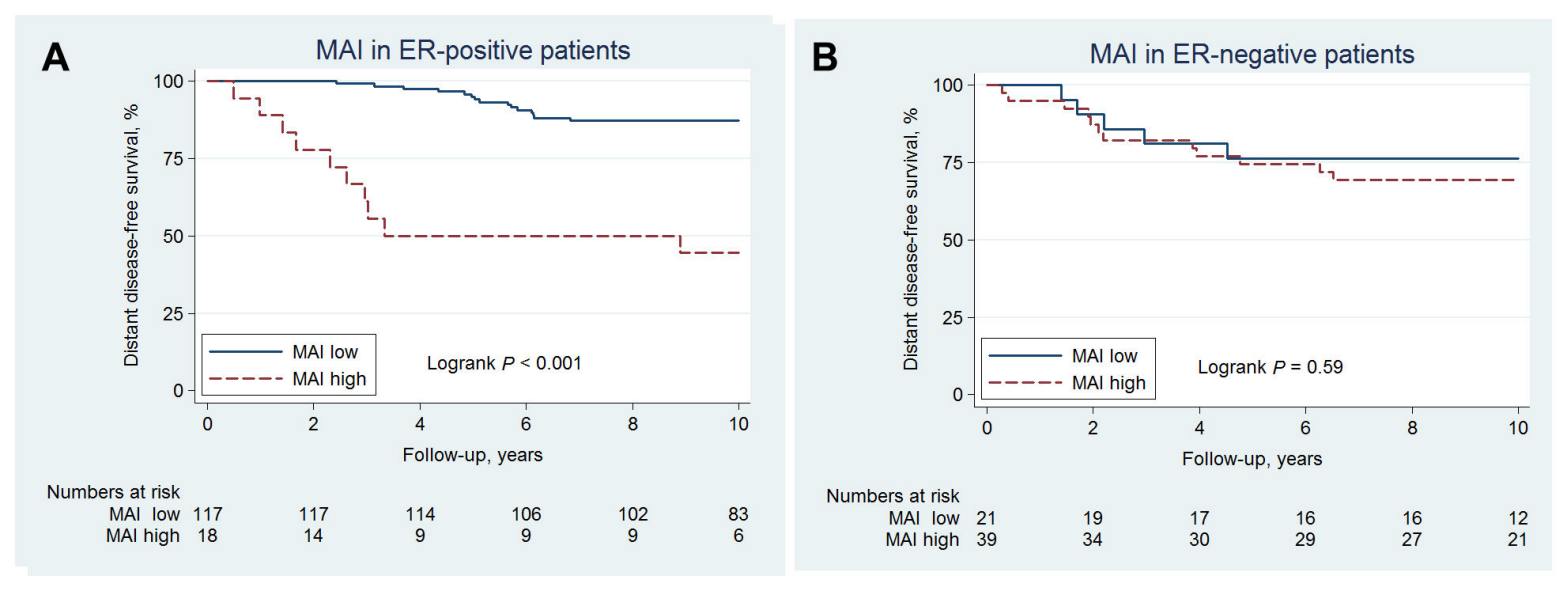
10-year distant disease-free survival of 195 premenopausal women with lymph-node negative breast cancer according to MAI-status in (a) ER-positive patients (b) ER-negative patients.

When stratifying for ER-status, a very strong negative effect of high MAI was found in ER-positive patients (HR=7.0, 95%CI: 3.1-16, *p*<0.001) with a 10-year DDFS of 44% (95%CI: 22-65) and 87% (95%CI: 80-92%) for high- and low-risk patients, respectively. No prognostic effect was found in ER-negative patients (HR=1.3, 95%CI: 0.47-3.8, *p*=0.59), Figure 2a-b. The prognostic effect of MAI in ER-positive and ER-negative patients was further analyzed and found to differ corresponding to a significant interaction term (HR=5.0, 95%CI: 1.3-19, *p*=0.017). Similar effects were found for PPH3, cyclin B1, cyclin A, and Ki67, Table 3. 

**Figure 3:**
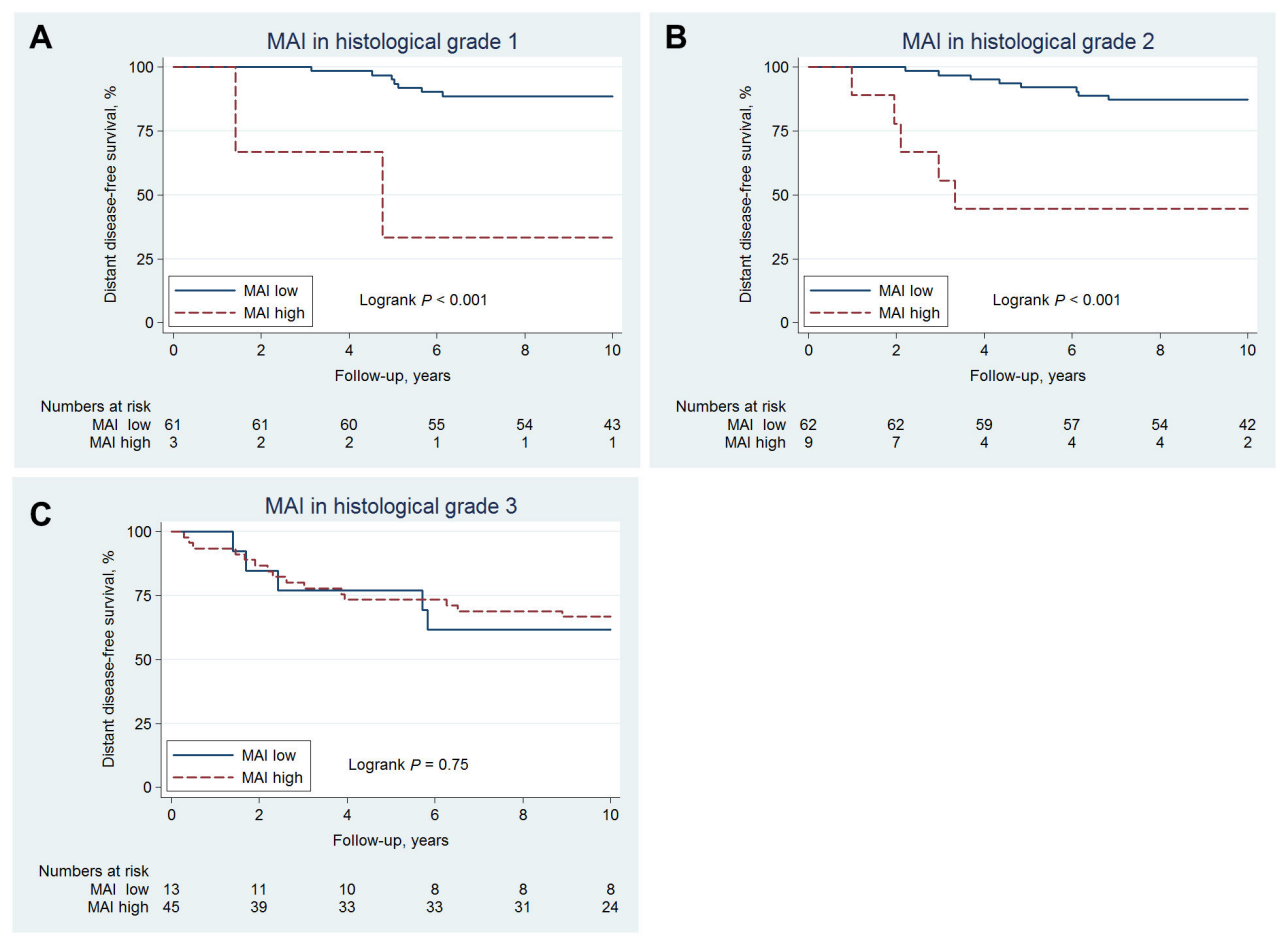
10-year distant disease-free survival of 195 premenopausal women with lymph-node negative breast cancer according to MAI-status in (a) histological grade 1 (b) histological grade 2 (c) histological grade 3.

For histological grade, no added prognostic value for MAI was found in histological 3. However, a strong added prognostic effect was found in histological grade 2 (HR=7.2, 95%CI: 3.1-22, *p*=0.001) and in grade 1 (HR=11, 95%CI: 2.3-55, *p*=0.003)*,* Figure 3a-c. Similar but weaker effects were found for PPH3, cyclin B1, cyclin A, and Ki67 (data not shown).

### Combinations of proliferation factors

**Figure 4:**
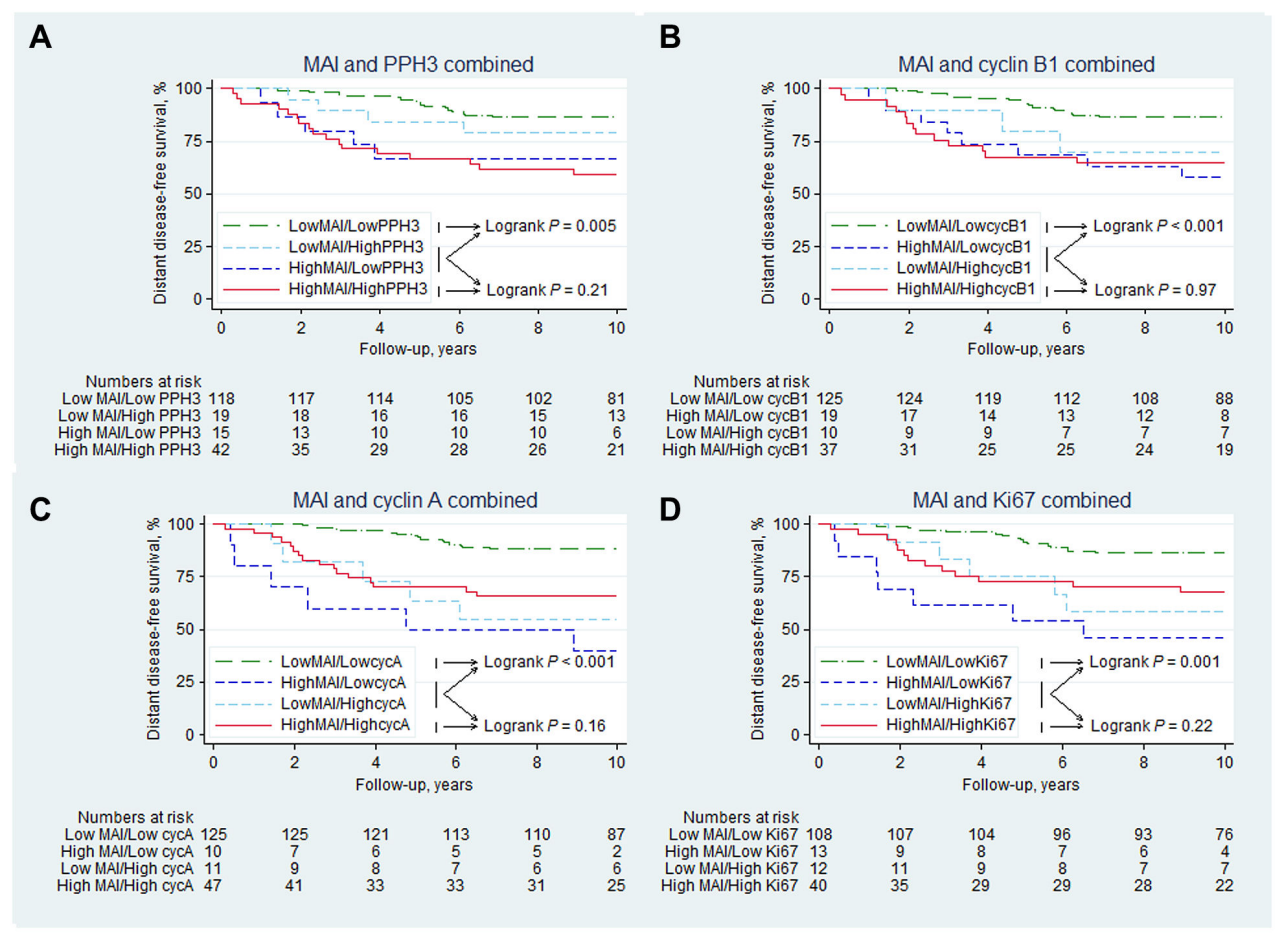
10-year distant disease-free survival of 195 premenopausal women with lymph-node negative breast cancer with data available on MAI, with MAI combined with (a) PPH3 (b) cyclin B1 (c) cyclin A, and (d) Ki67 with patients stratified into four groups, with either 0, 1, or 2 factors positive. As can be seen in all figures, patients with at least one of the two proliferations factors positive have a significantly higher risk of recurrence than patients negative for both factors, and no significant differences in recurrence rates compared to patients with both factors positive.

In a series of two-factor analyses one proliferation factor at a time was combined with MAI. Only patients who had data on both proliferation factors available for each two-factor analysis were included. Discordant cases for all combinations with MAI were found to have a significantly higher risk of recurrence than patients negative for both factors (*p*<0.001 for the combination MAI and cyclin A, log rank test), and no significant differences in distant recurrence rates compared to patients with both factors high, Figure 4a-d. Patients with at least one of the two proliferation factors positive were therefore defined as high-risk.

By combining MAI and cyclin A (N=193), the prognostic value was strengthened (HR=4.2 95%CI: 2.2-7.9, *p*<0.001), corresponding to a 10-year DDFS of 60% (95%CI: 48-71%) for the 35% high-risk patients (68/193), compared with 88% (95%CI: 81-93%) for the 65% low-risk patients (125/193). No such added prognostic value was found for combinations of MAI with PPH3, cyclin B1 or Ki67, Table 3 *.*

### Multivariable analyses

162 patients (35 events) had data available on all proliferation factors MAI, PPH3, cyclin B1, Ki67, and cyclin A. In multivariable analyses adjusted for age, ER-status, HER2-status, adjuvant medical treatment and one proliferation factor at a time, MAI (HR=2.7, 95%CI: 1.1-6.7, *p*=0.035), and cyclin A (HR=2.7, 95%CI: 1.2-6.0, *p*=0.019) added independent prognostic value, whereas cyclin B1, PPH3, and Ki67 were non-significant, Table 3. In further analyses, with the same adjustments as above, combinations of two proliferation factors were added to the multivariable models. Patients were defined as high-risk if at least one of the two proliferation factors was high. The combination of MAI and cyclin A resulted in a HR that was higher than for either factor alone (HR=3.8, 95%CI: 1.6-8.7, *p*=0.002), Table 4. When stratifying for ER-status, a strong negative prognostic effect was seen in ER-positive patients only (n=114), for MAI (HR=5.9, 95%CI: 2.4-15, *p*=0.001), followed by Ki67, and cyclin A. Cyclin B1 and PPH3 did not add any prognostic value in the ER-positive subgroup. Combining MAI with other proliferation factors did not result in a higher HR than the one found for MAI alone, Table 4 *.*

**Table 4:** Multivariable analyses of 10-year distant disease-free survival in all premenopausal patients with node-negative breast cancer where complete data on all proliferations factors was available (n=162, left), and in the ER-positive patients only (n=114, right).

		**All patients (n=162, 35 events)**			**ER-positive patients (n=114, 23 events)**		
**Factor**		**Hazard Ratio**	**95% Confidence Interval**	***p*-value**	**Hazard Ratio**	**95% Confidence Interval**	***p*-value**
**MAI**	high *vs* low	2.7	1.1-6.7	0.035	5.9	2.4-15	<0.001
**PPH3**	high *vs* low	1.4	0.66-3.1	0.37	1.8	0.70-4.8	0.22
**Cyclin B1**	high *vs* low	1.5	0.62-3.7	0.36	1.8	0.59-5.4	0.31
**Cyclin A**	high *vs* low	2.7	1.2-6.0	0.019	3.1	1.2-8.0	0.016
**Ki67**	high *vs* low	1.8	0.81-4.1	0.15	3.6	1.4-8.9	0.007
**MAI and/or PPH3 high**	high *vs* low	2.0	0.89-4.7	0.093	3.3	1.4-7.9	0.008
**MAI and/or cyclin B1 high**	high *vs* low	2.6	1.1-6.2	0.027	4.0	1.6-10	0.003
**MAI and/or cyclin A high**	high *vs* low	3.8	1.6-8.7	0.002	4.9	2.0-12	<0.001
**MAI and/or Ki67 high**	high *vs* low	2.9	1.3-6.8	0.013	4.7	1.9-11	0.001

All models are adjusted for age, ER-status (not in the ER-positive patients), HER2-status, and adjuvant medical treatment.

## Discussion

This study on premenopausal node-negative breast cancer patients with long-term follow up again proves the importance of proliferation, especially in ER-positive patients. As ER-negative patients have a worse prognosis, and in general a higher proliferation rate than the ER-positive patients [2,43], they would more often be offered chemotherapy as endocrine treatment is not an option. Studies have also shown that in ER-negative patients genes associated with immune response and the complement system are most important for prognosis, and that a good prognosis group could be found within this context for whom adjuvant chemotherapy may be avoided [44]. The majority of breast cancer patients are however ER-positive, and the main focus therefore lies in identifying ER-positive patients with a risk of recurrence sufficiently low to avoid extensive adjuvant treatment, and at the same time identify high-risk patients within a low-risk cohort. Studies on genetic profiling have revealed that the main common denominator in genetic profiles is proliferation genes, and it is within these genes the prognostic information lies [1]. Results from the two prospective studies on genetic profiles, MINDACT and TailorX have not yet been published, and in the 2011 St Gallen guidelines Ki67 is recommended as the surrogate proliferation marker of choice to distinguish between the low- and high-proliferative ER-positive luminal subtypes. However, there is a lack of consensus on assessment of Ki67 and choice of cut-off, and the reproducibility has been questioned [11]. Therefore, studies assessing other proliferation markers are needed.

The present study proves all included proliferation factors to be of prognostic value in the whole patient cohort, but more specifically in ER-positive patients. All factors stratified ER-positive patients into a low- and a high-proliferating group, a “luminal A”- and a luminal B”-like group, with significant differences in prognosis. No such effect was found in the ER-negative patients, which is probably due to the higher proliferation rates found in ER-negative patients [2,43]. 

MAI was the strongest prognostic proliferation factor, with as much as a three-fold hazard of distant recurrence at 10 years, both in univariable- and multivariable analysis, and a 7-fold hazard of distant recurrence in the ER-positive patients. MAI has the advantage, when it comes to clinical applicability, that evaluation of mitoses is already a part of routine histological grading. However, the protocol for assessment of MAI is more rigorous than mitosis assessment according to Elston and Ellis [39]. In the present study, when adhering to this stricter protocol of assessment of mitoses, the prognostic value of MAI surpasses histological grade and Ki67. This strong prognostic value of MAI confirms results from previous studies [13,15,18,20,41].

The other mitosis- and late G2-phase specific proliferation marker PPH3 also, as in previous studies [27–29], proved to be of strong prognostic value in all patients, and more specifically in ER-positive patients. PPH3 is a less extensively studied proliferation factor, and the staining is clear and contrast-rich, easily assessed, and with high inter-observer reproducibility [28]. Additionally, PPH3 does not stain apoptotic cells, which otherwise by routine assessment can be mistaken for mitotic cells. Lastly, PPH3 assessment only requires counting of positively stained cells, which is less time-consuming than assessment of Ki67. It could therefore be a support to MAI assessment. Similar to MAI, PPH3 and Ki67 values are higher in the periphery of the tumour, the growing zone, than in central less proliferative parts of the tumours. To facilitate comparisons between the different proliferation factors in the present study the predefined cut-offs chosen were the same for all factors assessed on TMA, the 7^th^ decile, which for PPH3 corresponded to ≥7 positive cells in 10 consecutive FOVs. In previous publications on PPH3, the optimized cut-off of ≥13 positive cells was found when assessing PPH3 on whole sections, with assessments starting at the periphery. This cut-off corresponded to >35% PPH3 positive tumours, quite similar to the 7^th^ decile chosen here [29]. In the present study, PPH3 was assessed on TMA cores which had been taken from both the periphery and the less proliferative centre of the tumour, which may explain why the same decile may correspond to different absolute cut-off values. Cyclin B1 also added prognostic value, although not as strong as MAI and PPH3.

Histological grade 2 patients constitute 30-60% of all patients, and they have a variable prognosis. We have previously shown that by stratifying grade 2 patients for another proliferation factor, Ki67, two groups with significant differences in prognosis were found, similar to grade 1 and grade 3 tumours, respectively [40]. These findings are in line with results for the genetic profile Genomic Grade Index, where one of the candidate genes is *KI67* [7]. Similar effects were found for grade 1 and 2 patients for all proliferation factors in the present study. However, as the numbers at risk with grades 1 and 2 and high MAI were few, the statistical power was low in these subgroup analyses, and results should be confirmed in larger patient series.

All single factors have their methodological advantages and disadvantages, and genetic profiles consist of multiple proliferation genes [1]. A recent study has also suggested that although all commercially available genetic profiles add independent prognostic value, the best prediction of recurrence was found if profiles were combined [9]. We therefore hypothesised that by combining two factors, of which at least one was positive, the prognostic value could be strengthened. We could show that for the entire patient cohort, not only patients with both factors positive, but also those with at least one factor positive (11-20% of all patients), had a significantly worse prognosis than patients negative for both factors. The strongest combination in univariable analysis in the present study was MAI combined with cyclin A. As cyclin A is expressed in S-phase, and MAI in M-phase, they might complement each other as the combination covers a greater span of the cell cycle. This is in line with a recent study by Gudlaugsson et al in which Ki67 yielded additional prognostic information in low proliferative breast cancers, with either MAI or PPH3 [45]. In the present study other combinations with MAI did not strengthen the prognostic value of MAI, but this may have been due to the limited number of patients and events in the present study. As all factors add prognostic value in univariable analysis it is likely that more combinations could have added prognostic strength in a larger patient set.

Lastly, an important advantage of immunohistochemical (IHC) assessments compared to genetic profiling, is the possibility of selective analysis of the invasive tumour only, excluding normal cells, in situ components, and areas of inflammation and necrosis, which could contaminate samples sent for genetic profiling. Also, it allows for selective assessment of proliferation in the periphery of the tumour where the proliferation rates are the highest. With standardisation of IHC procedures and factors that influence reproducibility, such as choice of detection system and antibody, cut-offs, and tissue section thickness, the quality of the IHC stains and reproducibility can be significantly improved [12,46].

In conclusion, the proliferation factors MAI, PPH3, and cyclin A are all of strong prognostic value in node-negative breast cancer, specifically in ER-positive patients and patients with histological grade 2. MAI, followed by PPH3, and cyclin A, was the strongest prognostic proliferation factor in the present study. This study also suggests that the prognostic value of proliferation is improved when combining two proliferation factors, in the present study MAI with cyclin A, and this can be used when deciding on risk and choice of adjuvant medical treatment.

### Ethical Standards

The study was approved by the ethics committee of Lund University Hospital (LU 240-01). The study protocol contained a written patient information sheet which was given to all patients and a written instruction for the doctor on how the information should be given to the patients. This was followed by verbal informed consent which was documented in the patients´ records. Written informed consent was not required by the Ethics Committee of Lund when this study was conducted, and the above mentioned procedure was preferred in national trials and approved by the ethics committee of Lund University Hospital prior to the initiation of the study.
